# Utility of a minimal skin incision laparotomy technique for removing uterine leiomyomas at a regional core hospital: a retrospective study

**DOI:** 10.1186/s13256-018-1703-2

**Published:** 2018-06-25

**Authors:** Ryo Sugiyama, Wataru Isono, Wada-Hiraike Osamu, Masanori Maruyama

**Affiliations:** 1 Department of Obstetrics and Gynaecology, Graduate School of Medicine, The University of Tokyo, 7-3-1, Hongo, Bunkyo-ku, Tokyo 113-0033 Japan; 2 Department of Obstetrics and Gynaecology, Maruyama Memorial General Hospital, 2-10-5, Motomachi, Iwatsuki-ku, Saitama-shi, Saitama 339-8521 Japan

**Keywords:** Minimal skin incision abdominal myomectomy, Blood loss, Operation time, Characteristics of leiomyoma

## Abstract

**Background:**

We present a minimal skin wound abdominal myomectomy performed in our hospital and attempt to identify the optimal range of this technique by considering the characteristics of target leiomyomas. In this procedure, we attempted to make the skin wound as small as possible, with a maximum length of approximately 5 cm.

**Methods:**

In addition to introducing the minimal skin wound abdominal myomectomy, we retrospectively collected and analyzed the medical records of 76 patients treated with minimal skin wound abdominal myomectomy exclusively by the same physician at Maruyama Memorial General Hospital between January 2007 and December 2016. We statistically investigated relationships between ten factors, including body mass index; patient’s age; patient’s parity; administration of gonadotropin-releasing hormone analogue; presence of anemia; the uterine leiomyomas’ number, size, weight, and location; operation time; and blood loss.

**Results:**

First, we introduce a case in which we performed minimal skin wound abdominal myomectomy for a 36-year-old Japanese patient with a large leiomyoma (10 cm in diameter). Then, we assessed the impacts of patient characteristics and leiomyoma characteristics on operation time and blood loss for this surgical method. In a multivariate analysis, only the number of resected leiomyomas significantly affected massive bleeding. Other factors showed no difference on operation time and the amount of blood loss.

**Conclusions:**

Minimal skin wound abdominal myomectomy is safe and effective for use in many patients, because only the number of leiomyomas affects the amount of blood loss. No other factor affected operation time. We suggest the possibility that the expanded use of minimal skin wound abdominal myomectomy may reduce the number of patients waiting for long periods to undergo laparoscopic surgery and may optimize the use of medical resources in rural areas.

## Background

Uterine leiomyomas are the most common estrogen-dependent benign tumors in women of reproductive age [1–4]. All gynecologists in local private clinics and major regional or university hospitals often encounter patients with this disease, and some of these patients will require operations. Because leiomyomas distort the uterine cavity, even relatively small ones may cause patients of reproductive age to have symptoms such as hypermenorrhea, decreased fertility, and increased miscarriage [5, 6] . The risk of perinatal diseases, premature delivery, and placental abruption is considered to be increased in these patients [7–9]. Therefore, many previous studies have concluded that large (over 5 cm in diameter) and multiple leiomyomas should be removed from women of reproductive age planning future pregnancy or childbirth [10, 11]. Other than uterine artery embolization and hysteroscopic myomectomy, which are performed for patients with submucosal leiomyomas [12, 13], almost all the interventions performed for women of reproductive age with leiomyomas are surgeries [14]. Compared with other gynecological surgeries, most target patients are relatively young and inevitably request laparoscopic surgery because of its advantages, including superior aesthetic results, faster recovery, and a shorter hospital stay [15, 16]. However, in addition to an increased risk of uterine wall damage during pregnancy caused by the difficulty of laparoscopic suturing procedures [17, 18], long operative times are typical disadvantages of laparoscopic myomectomy [19]. When sufficient hard and soft capacity are available at large hospitals, the choice of surgical technique depends directly on tumor characteristics, including size, number, and location as well as patient preferences. However, in Japan, most hospitals with an obstetrics and gynecology department, especially those in rural areas, have severely limited human resources because most gynecologists in Japan must manage deliveries. Under such circumstances, shorter operative times and fewer complications, especially massive blood loss, are much more important. When laparoscopic surgeries cannot be performed in their facilities, clinicians need to determine whether to refer patients to large hospitals with greater functionality. However, large hospitals have many patients waiting for surgical treatment because a large number of patients visit these hospitals, exceeding their capabilities. For women of advanced reproductive age who want to give birth, it is especially important to eliminate the long period between diagnosis and treatment to allow for the contraceptive period required after myomectomy. Thus, it is important to provide accurate and varied information to help patients select their treatment methods, although recently, most clinicians have begun to pay great attention to the advantages of laparoscopic surgeries or other cutting-edge technologies. Therefore, we introduce our minimal skin incision laparotomy technique for leiomyomas, which offers improvements on classical abdominal myomectomy. In this procedure, we attempted to make the abdominal wound as small as possible, with a maximum length of approximately 5 cm. In this retrospective analysis of patients who wished to preserve their uterus and underwent myomectomy, we tried to identify the optimal application of our surgical method in terms of decreased operative time and reduced blood loss.

## Methods

### Data collection in patients and predictors of operation time and blood loss

This study protocol was reviewed and approved by the human ethics committee of Maruyama Memorial General Hospital (reference number 2017-01). Subjects who underwent minimal skin incision abdominal myomectomy (MAM) at Maruyama Memorial General Hospital between January 2007 and December 2016 were included, and the medical records of 84 patients were reviewed retrospectively. This study included 76 patients who underwent MAM for the first time, all of whom were confirmed as having pathologically proven leiomyomas. We excluded patients who underwent other operations, such as ovarian cystectomy or hysteroscopic surgery, or those who had a congenital uterine abnormality or a malignant tumor. Data on body mass index (BMI; measured as kg/m^2^), age, patient’s parity, the administration of gonadotropin-releasing hormone analogue (GnRHa), and the presence of anemia were collected from the medical records. As predictors of the level of difficulty for each method, the number of leiomyomas and the size and location of the dominant leiomyoma were determined using magnetic resonance imaging and ultrasound before surgery. The location of the dominant leiomyoma was classified within two categories as follows: (1) posterior or two other types of leiomyomas, including anterior or fundal leiomyoma; and (2) subserous or two other types of leiomyomas, including intramural or submucous leiomyomas. The total weight of the resected leiomyomas from each patient was measured after surgery.

### Statistical analyses

The statistical analyses were performed using JMP version 12 for Windows (SAS Institute, Inc., Tokyo, Japan). The categorical variables of the measured characteristics of the leiomyomas and other factors were compared to determine the correlations between these characteristics and the operation time or blood loss. To eliminate confounding factors, we divided the patients into two groups according to the existence or nonexistence of each factor and used multivariate logistic regression analysis. For all patients treated with MAM, we assessed the influence of the following ten factors:

1. Higher BMI, defined as ≥ 25 kg/m^2^
2. Advanced age, defined as age ≥ 40 years
3. Anemia, defined as serum hemoglobin level < 10 g/dl
4. Multiparity, defined as a patient who has delivered at least once
5. GnRHa, defined as a patient who was administered GnRHa before surgery
6. Large leiomyoma, defined as a dominant leiomyoma ≥ 8 cm
7. Multiple leiomyomas, defined as three or more leiomyomas
8. Subserous leiomyoma, defined as a dominant subserous leiomyoma
9. Posterior leiomyoma, defined as dominant posterior leiomyoma
10. Heavy leiomyoma, defined as total weight of the resected leiomyomas ≥ 300 g

The criteria for “large leiomyoma” and “multiple leiomyomas” were determined on the basis of previous research [20, 21]. The ORs and 95% CIs were estimated to determine the strength of these correlations. *P* < 0.05 was considered statistically significant.

### Surgical technique: minimal skin incision abdominal myomectomy

**Fig. 1:**
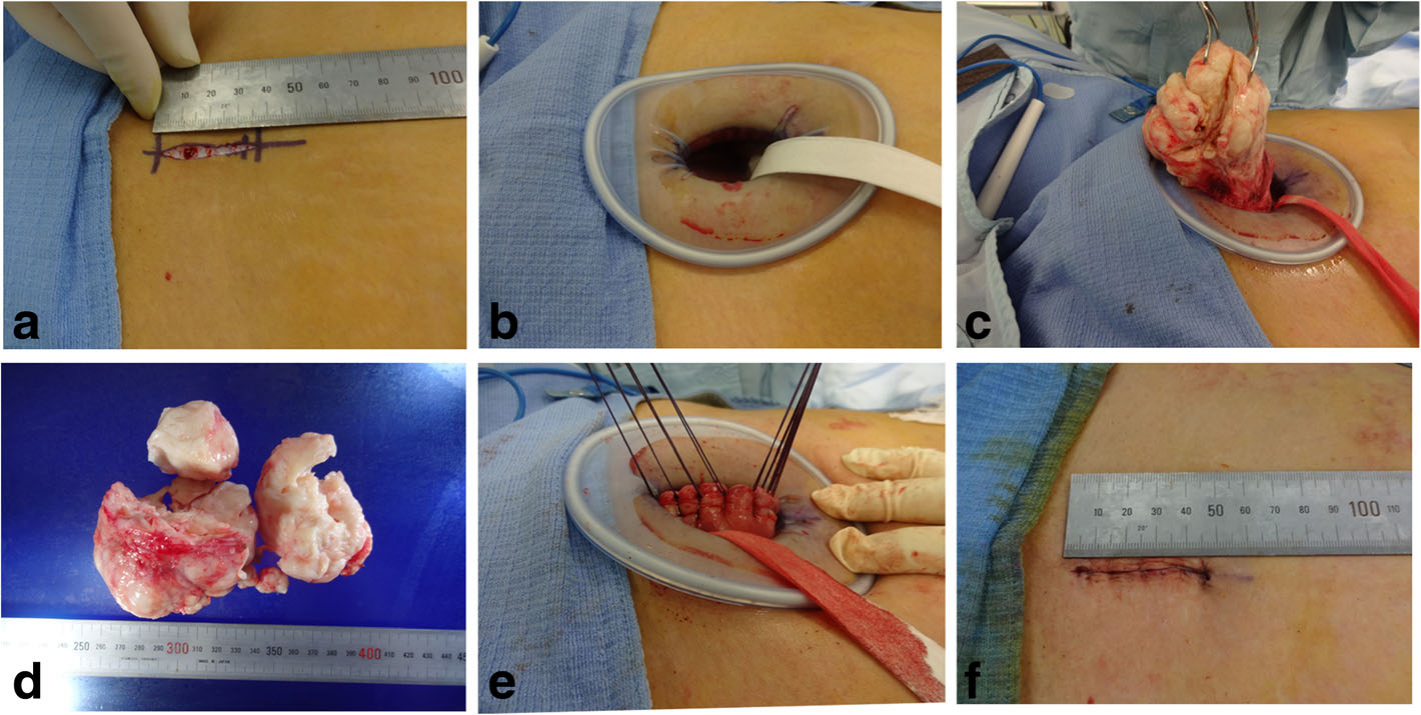
Surgical procedures of minimal skin incision abdominal myomectomy. This patient was a 36-year-old woman with two uterine leiomyomas. The larger leiomyoma was 10 cm in diameter on admission, and it decreased to 8 cm in diameter after two doses of gonadotropin-releasing hormone analogue. The patient had a history of gravida 2 para 1. The operation time was 75 minutes, and the patient’s blood loss was 28 ml. The incision site was 3.5 cm. We extracted two leiomyomas from the site. The total weight of the leiomyoma was 234 g. **a** The size of the skin incision. **b** The appearance of the wound with the Small Alexis® Wound Protector/Retractor. **c** The leiomyoma was grasped with sharp clamps and pulled through the wound after some cuts were made and the diameter of leiomyoma was reduced. **d** The appearance of the resected leiomyomas. **e** The uterine wound was sutured with layered sutures. **f** The appearance of the sutured skin wound

All patients were operated on in the lithotomy position under general endotracheal anesthesia, and a Foley catheter was placed inside the bladder. Epidural anesthesia was also added. A skin incision less than approximately 5 cm in length was made longitudinally (4.3 ± 0.7 cm, 3–5.5 cm, *n* = 76), and the adipose tissue and abdominal fascia were cut using a monopolar electric scalpel (Fig. 1a). After the rectus abdominis muscle was spread, the peritoneal membrane was opened with a scalpel and surgical scissors. In this procedure, the abdominal fascia and peritoneal membrane were cut longitudinally at a length of approximately 6 cm. A Small or Medium Alexis® Wound Protector/Retractor (Applied Medical Resources Corporation, Rancho Santa Margarita, CA, USA) was placed inside the wound to provide a wide operative view, making the uterus visible (Fig. 1b). Vasopressin solution was injected into the surrounding tissue to decrease bleeding after the locations of the leiomyomas were detected. The surface of the uterine trunk was cut with a monopolar electric scalpel, and a part of the leiomyoma was grasped with a sharp clamp. While pulling the leiomyoma through the wound in an upward direction, a surgeon cut part of it to remove it from the wound and then grasped a different location (Fig. 1c). By repeating these procedures, even leiomyomas that were larger than the wound could be removed (Fig. 1d). The uterine wound was sutured with layered sutures (Fig. 1e), and then the peritoneum, fascia, and skin were sutured (Fig. 1f).

## Results

### Characteristics of patients treated with MAM

**Fig. 2:**
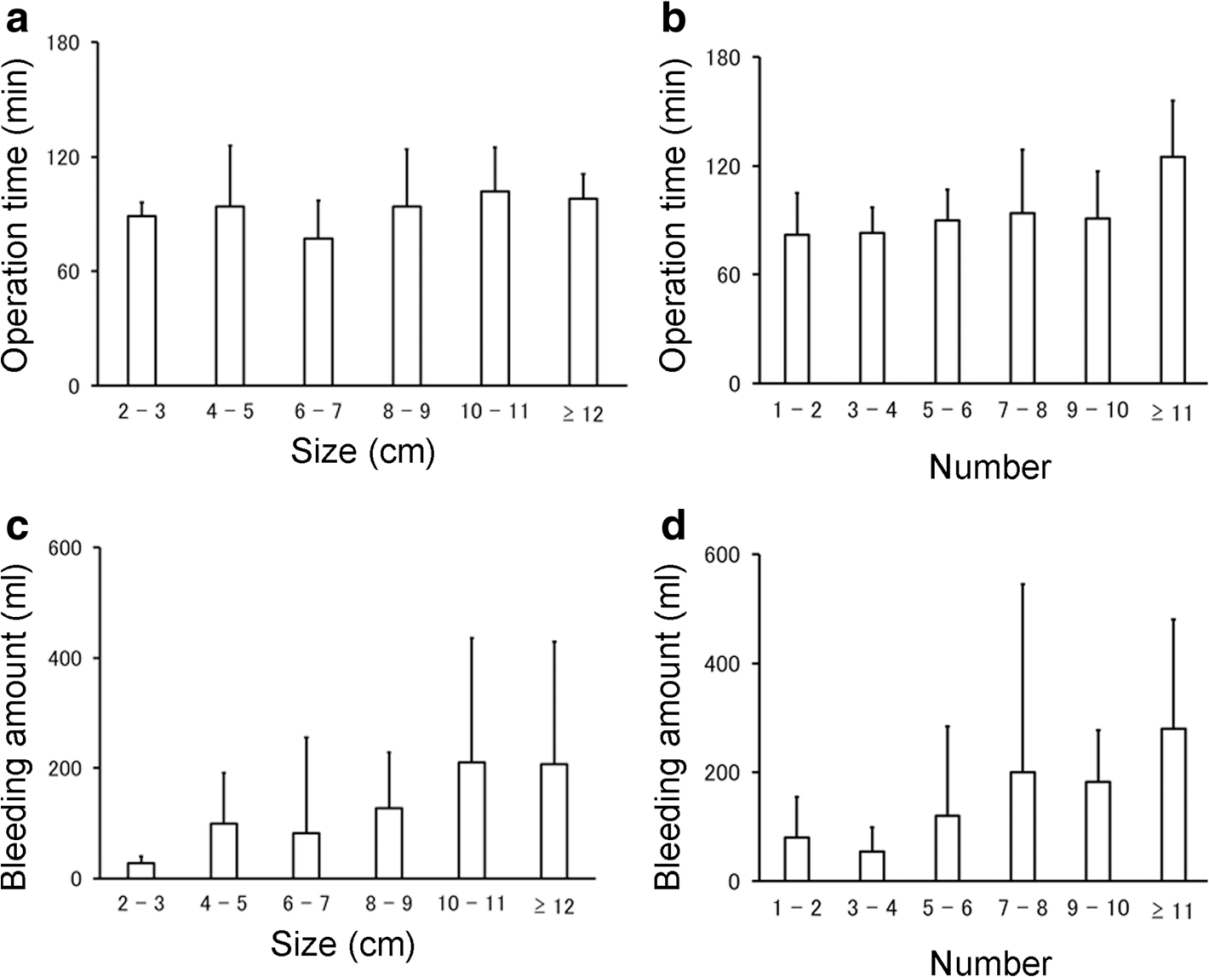
The influence of leiomyoma size and number on the difficulty of myomectomy. To assess the relationship between the level of difficulty of minimal skin incision abdominal myomectomy and the characteristics of leiomyoma, we extracted the size of dominant leiomyoma determined with diagnostic imaging (**a**, **c**) or the number of resected leiomyomas (**b**, **d**) and divided all patients into six groups. The average operation time (**a**, **b**) or amount of bleeding (**c**, **d**) was calculated in these six groups. The standard deviation (SD) in these groups was also indicated. Apart from the relationship between the size of leiomyoma and operation time (**a**), we detected a tendency of operation time and blood loss to increase as the size and number of leiomyomas increased (**b**–**d**). The number of patients in each group divided by the size and number of leiomyomas are as follows: Size: 2 to 3 cm, *n* = 2; 4 to 5 cm, *n* = 17; 6 to 7 cm, *n* = 24; 8 to 9 cm, *n* = 17; 10 to 11 cm, *n* = 9; over 12 cm, *n* = 7. Number: 1 to 2, *n* = 30; 3 to 4, *n* = 13; 5 to 6, *n* = 15; 7 to 8, *n* = 6; 9 to 10, *n* = 3; over 11, *n* = 9

**Fig. 3:**
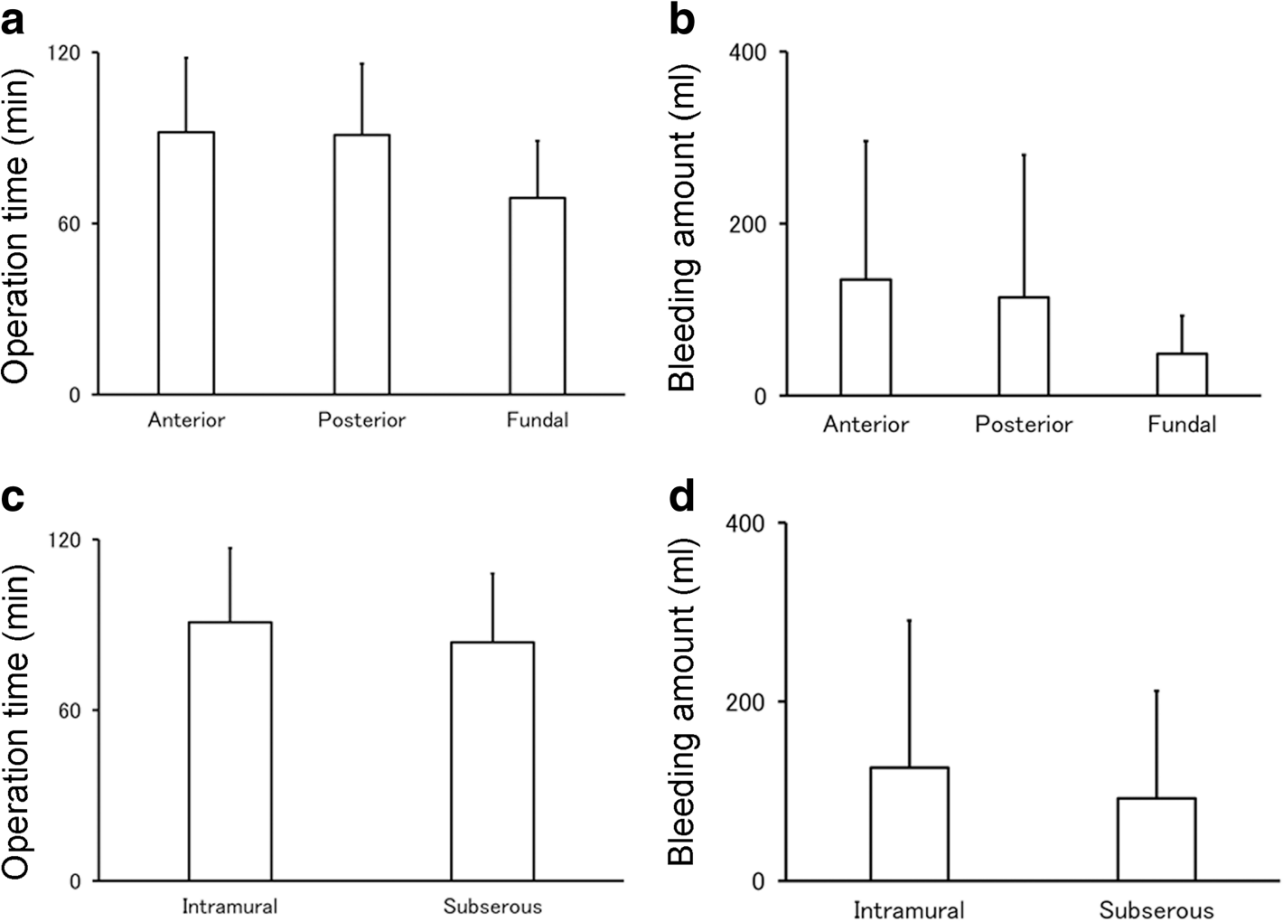
The influence of leiomyoma location on the difficulty of myomectomy. To assess the relationship between the level of difficulty of minimal skin incision abdominal myomectomy and the characteristics of leiomyoma, we extracted the location of dominant leiomyoma as determined with diagnostic imaging. Target leiomyomas were classified into the two types as follows: anterior, posterior, and fundal leiomyomas (**a**, **b**) and intramural, subserous, and submucous leiomyomas (**c**, **d**). There was no patient whose dominant leiomyoma was a submucous leiomyoma. The location of dominant leiomyomas had no significant impact on the average operation time (**a**, **c**) or amount of bleeding (**b**, **d**). However, fundal leiomyomas showed a tendency of shorter operation time than anterior leiomyomas (*P* = 0.068) or posterior leiomyomas (*P* = 0.083). The number of patients in each group divided by the location of dominant leiomyomas was as follows: **a**, **b**: Anterior leiomyoma, *n* = 40; posterior leiomyoma, *n* = 31; fundal leiomyoma, *n* = 5. **c**, **d**: Intramural leiomyoma, *n* = 64; subserous leiomyoma, *n* = 12

The average age of patients treated with MAM was 37.1 ± 4.6 years, and approximately 80% of all patients (60 of 76) were treated with GnRHa before undergoing surgery. The average BMI was 22.0 ± 2.8 kg/m^2^. The average diameter of the dominant leiomyoma was 7.5 ± 2.7 cm (range, 3–15 cm), and average number of resected leiomyomas was 5.3 ± 5.7 (range, 1–28). The average total weight of resected leiomyomas was 174 ± 159 g (range, 4–868 g). The overall average operation time in 76 patients was 90 ± 26 (40–181) minutes, and the overall average blood loss was 121 ± 159 (0–877) ml. First, we divided 76 patients into 6 groups by the size of dominant leiomyoma (2 to 3 cm, 4 to 5 cm, 6 to 7 cm, 8 to 9 cm, 10 to 11 cm, and over 12 cm) and by the number of resected leiomyomas (1 to 2, 3 to 4, 5 to 6, 7 to 8, 9 to 10, over 11). Then, we examined the relationship between the characteristics of the leiomyoma and the difficulty of the operation. As shown in Fig. 2a–d, visually both operation time and blood loss increased as the size and number of leiomyomas increased. However, when comparing the amount of blood loss (Fig. 2c and d), we found that operation time was not easily affected by the size and number of leiomyomas (Fig. 2a and b). The location of the dominant leiomyoma, including two types of classification, did not significantly affect either operation time or the amount of blood loss (Fig. 3a–d). Among the leiomyomas, fundal leiomyoma showed a tendency of shorter operation time than anterior (*P* = 0.068) and posterior leiomyomas (*P* = 0.083) (Fig. 3a).

To detect the practically significant factors affecting the complexity of MAM, we extracted information about the characteristics of leiomyoma. The size, number, and location of leiomyomas were searched before surgery, and the total weight of resected leiomyomas was measured after surgery (Table 1). BMI, age, and parity were also taken into consideration because the amount of subcutaneous fatty tissue directly affected operative view. “Long-duration operation” and “massive bleeding” were defined as 120 minutes and 275 ml, respectively, based on the average and standard deviation (SD) values of 76 patients. In multivariate analysis for these ten factors, only “multiple leiomyomas” significantly affected the amount of blood loss (OR = 5.2, *P* < 0.05) (Table 1). This factor also had a tendency of increasing the possibility of the factor “Long-duration operation” (OR = 6.1, *P* = 0.062) (Table 1). However, the size and weight of the resected leiomyomas did not show a significant difference.

**Table 1:** Risk of increasing level of difficulty of minimal skin incision abdominal myomectomy

		Long-duration operation		Massive bleeding	
Risk factors	Number	OR (95% CI)	*P* value	OR (95% CI)	*P* value
Large leiomyoma	33	1.7 (0.6–4.7)	NS	2.4 (0.7–7.6)	NS
Multiple leiomyoma	46	6.1 (0.6–62.4)	NS	5.2 (0.5–55.0)	*P* < 0.05
Subserosal leiomyoma	12	0.6 (0.1–7.1)	NS	0.7 (0.1–8.6)	NS
Posterior leiomyoma	31	0.4 (0.1–1.5)	NS	0.9 (0.3–2.7)	NS
Heavy leiomyoma	13	2.9 (0.9–9.7)	NS	2.9 (0.9–9.7)	NS
Higher BMI	12	1.6 (0.4–7.2)	NS	1.9 (0.4–9.0)	NS
Advanced age	25	2.9 (1.1–8.2)	NS	0.7 (0.2–2.7)	NS
Anemia	9	0.9 (0.1–11.0)	NS	Impossible to calculate	NS
Multiparity	10	0.8 (0.1–9.4)	NS	0.9 (0.1–11.3)	NS
GnRHa	60	2.3 (0.2–24.8)	NS	2.0 (0.2–22.0)	NS

*Abbreviations: BMI* Body mass index, *GnRHa* Gonadotropin-releasing hormone analogue, *OR* Odds ratio, *CI* Confidence interval, *NS* Not significant

Ten factors were defined as follows: (1) “large leiomyoma,” defined as a dominant leiomyoma ≥ 8 cm; (2) “multiple leiomyomas,” defined as three or more leiomyomas; (3) “subserous leiomyoma,” defined as a dominant subserous leiomyoma; (4) “posterior leiomyoma,” defined as dominant posterior leiomyoma; (5) “heavy leiomyoma,” defined as total weight of the resected leiomyomas ≥ 300 g; (6) “higher BMI,” defined as BMI ≥ 25 kg/m^2^; (7) “advanced age,” defined as age ≥ 40 years; (8) “anemia,” defined as serum hemoglobin level < 10 g/dl; (9) “multiparity,” defined as a patient who has delivered at least once; and (10) “GnRHa,” defined as a patient who was administered GnRHa before surgery. The relationship between “long-duration operation” or “massive bleeding,” which were defined as 120 minutes or 275 ml, respectively, and these ten factors was assessed by multivariate analysis. In this analysis, only “multiple leiomyomas” had the significant impact on the amount of blood loss (*P* < 0.05). “Multiple leiomyomas” had also the tendency of increasing the risk of “long-duration operation” (*P* = 0.062) and “higher BMI” showed a tendency of increasing the risk of “massive bleeding” (*P* = 0.068)

## Discussion

The current trend is for laparoscopic surgeries to be performed mainly as myomectomy with the purpose of preserving the uterus, because faster recovery and a shorter hospital stay are expected in laparoscopic surgeries. In addition, surgical wounds after performing laparoscopic surgeries are smaller than those after abdominal surgeries [22]. However, as often happens in rural areas, laparoscopic surgeries cannot be performed owing to the shortage of skilled staff and insufficient capacity to always provide blood transfusions [23]. In our hospital (Maruyama Memorial General Hospital), which is located in one of the rural areas of Japan, abdominal surgeries are performed primarily for treating leiomyomas, and therefore we describe our procedure in this report. Because myomectomy is inevitably performed for treating young female patients who want to preserve their fertility, the best efforts should be made to minimize the abdominal wound, even though laparoscopic surgeries cannot be performed. Although some skill is required, this operation could be introduced in facilities without new equipment.

Hospitals should always provide for the possibilities of relatively longer operations and massive blood loss, which are very important indexes of surgical difficulty. As mentioned above, it is very important to select the optimal surgery promptly, from a viewpoint of safety, human resources, and facilities. We aimed to find out which cases were troublesome and to look for the optimal range of this surgery. In this research, more than 2 hours of operation time was needed, as a practical matter, to perform MAM in only nine cases, namely “long-duration operation.” Over 275 ml of blood loss was regarded as massive bleeding and was detected in eight cases, but over 500 ml of blood loss occurred in only four cases. The influence of five factors related to the characteristics of target leiomyomas detected before surgery were mainly assessed. The results of multivariate analysis showed the disadvantage that the level of difficulty increased with increasing number of leiomyomas. However, the size and location had no significant influence on both the risk of long-duration operation and massive bleeding. In other words, there may be an advantage in performing MAM effectively and safely without relying on the characteristics of target leiomyomas when choosing this technique. These results could not be generalized simply, because there were many differences in the selection of target patients, surgeons performing myomectomy, and so forth. However, our analysis indicated the characteristics of MAM concisely, because all operations were performed exclusively by the same physician.

According to some past reports [21, 24] and the guidelines of the Japan Society of Gynecologic and Obstetric Endoscopy and Minimally Invasive Therapy (http://c-linkage.com/for/jsgoe/kaiin/pdf/comment.pdf), the size, number, and location of target leiomyomas should be considered when determining whether to perform laparoscopic myomectomy; however, only the number of resected leiomyomas affected the difficulty of MAM significantly (Table 1). This difference showed the possibility that the criteria for choosing this operational technique could be set relatively wide without taking the characteristics of target leiomyomas into consideration. This wide application criterion can be effective for many hospitals in rural areas. In consideration of the time and labor needed for introducing other facilities, this abdominal surgery reduces the loss of precious time caused by waiting for long periods to undergo laparoscopic surgery. Actually, 13 of 76 patients (17%) in our study received infertility treatment. The core of the operational technique introduced in this research was that a large leiomyoma was pulled from the small skin wound after making some cuts and reducing the diameter of this leiomyoma. The large leiomyoma became close to a linear shape when pulled out of the wound (Fig. 1c). Because this technique can be applied in other abdominal surgeries, including abdominal total hysterectomy, and because the size of skin wound can be reduced by removing large leiomyomas before starting to resect the uterus, further improvements should be expected.

## Conclusions

MAM is effective and safe for use in almost all cases because unlike other surgical methods, in MAM, operation time and blood loss are not influenced by almost all of the characteristics of target leiomyomas. The expanded use of MAM may optimize the use of medical resources in rural areas.

## Abbreviations

BMI: Body mass index
GnRHa: Gonadotropin-releasing hormone analogue
MAM: Minimal skin incision abdominal myomectomy
